# Protection of Human Umbilical Vein Endothelial Cells against Oxidative Stress by MicroRNA-210

**DOI:** 10.1155/2017/3565613

**Published:** 2017-03-07

**Authors:** Tianyi Li, Xianjing Song, Jichang Zhang, Lei Zhao, Yongfeng Shi, Zhibo Li, Jia Liu, Ning Liu, Youyou Yan, Yanlong Xiao, Xin Tian, Wei Sun, Yinuo Guan, Bin Liu

**Affiliations:** Department of Cardiology, The Second Hospital of Jilin University, Changchun, Jilin, China

## Abstract

Oxidative stress induces endothelial cell apoptosis and promotes atherosclerosis development. MicroRNA-210 (miR-210) is linked with apoptosis in different cell types. This study aimed to investigate the role of miR-210 in human umbilical vein endothelial cells (HUVECs) under oxidative stress and to determine the underlying mechanism. HUVECs were treated with different concentrations of hydrogen peroxide (H_2_O_2_), and cell viability was evaluated using the 3-(4,5-dimethylthiazol-2-yl)-2,5-diphenyltetrazolium bromide assay and ATP assay. To evaluate the role of miR-210 in H_2_O_2_-mediated apoptosis, gain-and-loss-of-function approaches were used, and the effects on apoptosis and reactive oxygen species (ROS) level were assayed using flow cytometry. Moreover, miR-210 expression was detected by quantitative reverse transcriptase polymerase chain reaction (qRT-PCR), and expression of the following apoptosis-related genes was assessed by qRT-PCR and Western blot at the RNA and protein level, respectively: caspase-8-associated protein 2 (CASP8AP2), caspase-8, and caspase-3. The results showed that H_2_O_2_ induced apoptosis in HUVECs in a dose-dependent manner and increased miR-210 expression. Overexpression of miR-210 inhibited apoptosis and reduced ROS level in HUVECs treated with H_2_O_2_. Furthermore, miR-210 downregulated CASP8AP2 and related downstream caspases at protein level. Thus, under oxidative stress, miR-210 has a prosurvival and antiapoptotic effect on HUVECs by reducing ROS generation and downregulating the CASP8AP2 pathway.

## 1. Introduction

Atherosclerosis is the leading cause of coronary heart disease, with high mortality and morbidity rates [1]. Under oxidative stress, excessive reactive oxygen species- (ROS-) induced endothelial cell apoptosis plays a crucial role in atherosclerosis development [2]. Therefore, reduction of endothelial apoptosis is vital in atherosclerosis prevention.

The association of microRNAs (miRNAs), which are involved closely in various cell processes through posttranscriptional downregulation of their target mRNAs, with apoptosis has drawn much attention [3–6]. In particular, expression of miRNA-210 (miR-210) has been considered a general response to hypoxia [7], and miR-210 has been reported to be participated in various cellular processes, including cell cycle arrest [8], angiogenesis [6], and cell survival [5] in various cell types, including endothelial cells [6]. Besides hypoxia, miR-210 is involved in oxidative stress-induced injury. Our previous study has revealed that miR-210 protects cardiomyoblasts against apoptosis under oxidative stress [9]. However, the role of miR-210 in cell apoptosis apparently varies with cell types and stimuli [10–13]. It has been reported that miR-210 protects cardiomyocytes against apoptosis under hypoxic conditions [10] and protects stem cells against oxidative stress [11]. However, some studies identified miR-210 as a proapoptotic component in human pulmonary artery epithelial cells [12] and in a human lung cancer cell line [13]. Therefore, the role of miR-210 in apoptosis in different cell types and under different conditions needs further investigation.

Some studies have indicated that miR-210 exerts antiapoptosis action by downregulating caspase-8-associated protein 2 (CASP8AP2) in stem cells under hypoxic conditions [5]. CASP8AP2 (or FLICE-associated huge protein), a 1,962-amino acid-long protein, takes part in several cellular processes, such as mRNA processing, cell cycle, apoptosis, and transcriptional control [14]. CASP8AP2 was identified as a proapoptosis member that acts via binding the death effector domains of procaspase-8, which activates caspase-8 and its downstream caspases, including caspase-3, thereby participating in the extrinsic apoptosis pathway mediated by CD95 [15, 16].

However, the effect of miR-210 on endothelial cells under oxidative stress remains unclear. In this study, we investigated the effects of miR-210 on apoptosis and the underlying mechanism in human umbilical vein endothelial cells (HUVECs) treated with hydrogen peroxide (H_2_O_2_), which has been commonly used to induce oxidative stress in in vitro models [17]. We found that miR-210 protected HUVECs against oxidative stress by reducing ROS generation and downregulating the CASP8AP2 pathway.

## 2. Materials and Methods

### 2.1. Cell Culture

HUVECs were obtained from the Department of Cardiology of The Second Hospital of Jilin University and cultured in a humidified incubator at 5% CO_2_ and 37°C. The complete growth medium for this cell line consisted of the vascular cell basal medium (ATCC, USA) with the endothelial cell growth kit-BBE (ATCC, USA) and contained the following components: 2% fetal bovine serum, 0.2% bovine brain extract, 10 mmol/L L-glutamine, 1 *μ*g/mL hydrocortisone hemisuccinate, 5 ng/mL rhEGF, 50 *μ*g/mL ascorbic acid, and 0.75 units/mL heparin sulfate.

### 2.2. Lentivirus Infection of HUVECs

A replication-deficient lentivirus was purchased from Sangon Biotech (Shanghai, China), encoding the human miR-210 precursor (pre-210), miR-210 inhibitor (anti-miR-210), or miR-scramble (miR-Scr, without miR-210 as a control) labeled with green fluorescent protein. 5 *μ*L of lentiviral vectors was added per well for transduction of HUVECs in 6-well plates (1 × 10^5^ cells/well) for 12 h. Fresh complete growth medium was subsequently added to replace the medium. To eliminate nontransduced cells, puromycin (Sigma-Aldrich, USA) was added to the medium at a final concentration of 4 *μ*g/mL [18], and the medium was incubated for at least 72 h. The transduced cells were then stained with DAPI and observed with a fluorescence microscope. The Image J software was used to quantitate the transduction efficiency.

### 2.3. DAPI Staining

After transduction with the lentivirus, cells were stained with DAPI to visualize the nucleus. Cells were cultured in a 6-well plate and fixed in 4% paraformaldehyde for 25 min at room temperature (25°C). Subsequently, cells were incubated with DAPI for 10 min and washed with PBS thrice.

### 2.4. Transfection

HUVECs were transfected in 6-well plates using Lipofectamine 2000 Reagent (Invitrogen, Carlsbad, CA, USA) according to the manufacturer's instructions. HUVECs were transfected with miR-210 mimics, miR-210 inhibitor, or negative control (NC) (GenePharma, Shanghai, China) at approximately 80% confluence.

### 2.5. 3-(4,5-Dimethylthiazol-2-yl)-2,5-diphenyltetrazolium Bromide (MTT) Detection

MTT detection was used to evaluate the viability of HUVECs treated with different concentrations of H_2_O_2_ and the effect of miR-210 on cell viability. Cells in a 96-well plate (1 × 10^4^ cells/well) were treated with different concentrations of H_2_O_2_ (0, 0.1, 0.2, 0.5, 1.0, 1.5, and 2.0 mmol/L) for 24 h. MTT (Biosharp, China) was added to each well, and the wells were incubated for 4 h at 37°C. Subsequently, the medium was removed, and dimethyl sulfoxide (Sigma-Aldrich, USA) was added (150 *μ*L/well) to solubilize the formazan crystals. Optical density was measured at 570 nm with a Varioskan Flash microplate reader (Thermo Scientific, USA), and cell viability was calculated as a percentage of the control optical density.

### 2.6. ATP Assay

To further evaluate cell viability, a luminescent ATP assay kit, CellTiter-Glo® Luminescent Cell Viability Assay (Promega, USA), was employed. Cells were treated with different concentrations of H_2_O_2_ (0, 0.1, 0.2, 0.5, 1.0, 1.5, and 2.0 mmol/L) for 24 h in a white 96-well plate (1 × 10^4^ cells/well). After the treatment, 100 *μ*L of CellTiter-Glo Reagent was added per well. The luminescence was then detected with a Varioskan Flash microplate reader (Thermo Scientific, USA). Cell viability was calculated as a percentage normalized to the control group.

### 2.7. Flow Cytometry

To detect apoptosis and determine ROS level of HUVECs treated with different concentrations of H_2_O_2_ and to assess the effect of miR-210, flow cytometry experiments were performed with the FACSCalibur system (BD Biosciences, USA). After treatment with different concentrations of H_2_O_2_ (0, 0.5, and 1.0 mmol/L), PE Annexin V Apoptosis Detection kit I (BD Biosciences, USA) was used following the protocol for apoptosis detection. After transfection with miR-210 mimics, miR-210 inhibitor, or negative control, cells were dealt with H_2_O_2_ (0.5 mmol/L). A Reactive Oxygen Species Assay Kit (Beyotime, China) was used following the protocol for ROS detection.

### 2.8. Quantitative PCR

Total RNA was extracted using Eastep® Super Total RNA Extraction Kit (Promega, USA) following the protocol. The quality of the extracted RNA was evaluated with a NanoDrop 2000 Spectrophotometer (Thermo Scientific, USA). To detect miR-210 expression levels, RNA was reverse-transcribed into cDNA by using the miRcute miRNA First-Strand cDNA Synthesis Kit (TIANGEN Biotech, China) according to the protocol. To determine expression profiles of apoptosis-related genes, RNA was reverse-transcribed into cDNA by using the TransScript One-Step gDNA Removal and cDNA Synthesis SuperMix Kit (TransGen Biotech, China) following the manufacturer's instructions. Thereafter, qRT-PCR was performed in the LightCycler 480 system (Roche, Switzerland) with TransStart Green qPCR SuperMix (TransGen Biotech, China). U6 was selected as the reference gene. qRT-PCR conditions were set as follows: 95°C for 1 min followed by 40 cycles of 95°C for 20 s and 61°C for 31 s. All primers were purchased from Sangon Biotech, China.

### 2.9. Western Blot

After 24 h of treatment with H_2_O_2_ (0 or 0.5 mmol/L), HUVECs were lysed using the radioimmunoprecipitation assay buffer with protease inhibitors, 1 mM phenylmethylsulfonyl fluoride, and mercaptoethanol. The homogenate was centrifuged for 15 min at 4,500 rpm at 4°C, and the supernatant was used for sodium dodecyl sulfate polyacrylamide gel electrophoresis at 20 *μ*g of total protein per lane, followed by transfer to a polyvinylidene fluoride membrane. The membranes were blocked with nonfat dry milk for 90 min at room temperature and incubated with primary monoclonal rabbit anti-CASP8AP2 (1 : 200; Santa Cruz Biotechnology, USA), monoclonal rabbit anti-caspase-8 (1 : 1000; Cell Signaling Technology, USA), monoclonal rabbit anti-caspase-3 (1 : 1000; Cell Signaling Technology, USA), and monoclonal mouse anti-*β*-actin (1 : 2000; Proteintech, USA) antibodies, followed by incubation with a secondary horseradish peroxidase-conjugated anti-rabbit IgG antibody or anti-mouse IgG antibody (1 : 1000; Beyotime, China). The blots were visualized using the SuperSignal West Pico kit (Thermo Scientific, USA). The relative expression level of each protein was normalized to that of *β*-actin.

### 2.10. Statistical Analysis

Data are reported as means ± SE. Statistical analysis was performed using SPSS13.0 (IBM, USA). For all experiments, Student's *t*-test was employed to assess statistical significance, and the threshold was set at *p* = 0.05.

## 3. Results

### 3.1. The Effect of H_2_O_2_ on Cell Viability and miR-210 Expression in HUVECs

**Figure 1:**
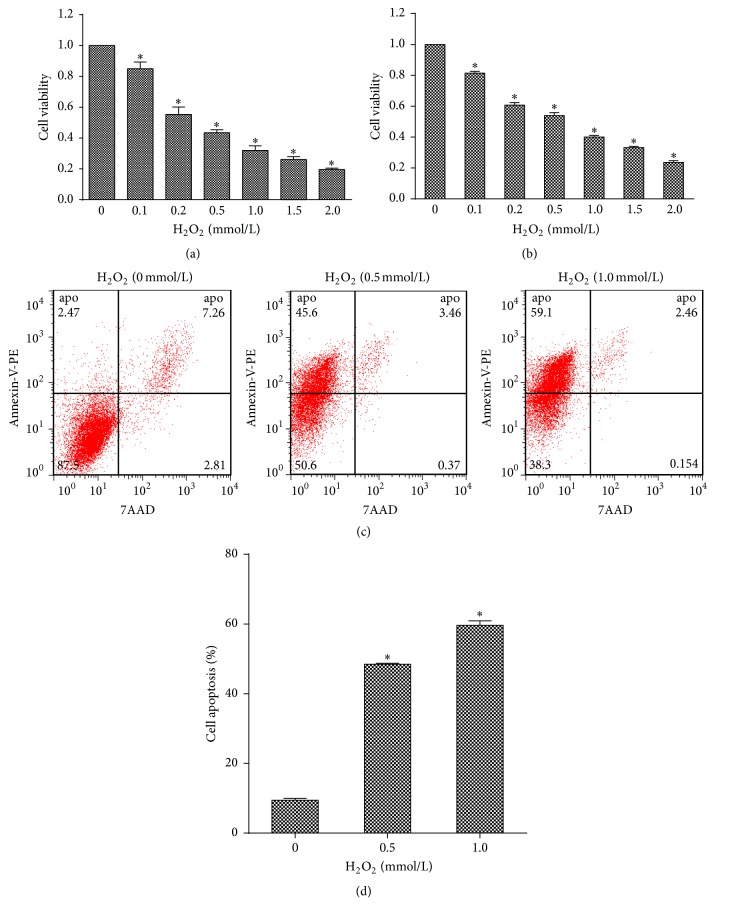
H_2_O_2_ induces cell death in a dose-dependent manner. (a) The effect of H_2_O_2_ on HUVECs viability compared to untreated control cells shown by the MTT assay. (b) Evaluation of HUVECs viability under H_2_O_2_ treatment using the ATP assay. (c) HUVECs were treated with 0, 0.5, or 1.0 mmol/L H_2_O_2_ for 24 h, and apoptosis was assessed by flow cytometry. (d) Flow cytometry analysis showed that H_2_O_2_ induced HUVEC apoptosis in a dose-dependent manner. All *n* = 3, ^*∗*^*p* < 0.05.

HUVECs were treated with different concentrations of H_2_O_2_ (0, 0.1, 0.2, 0.5, 1.0, 1.5, and 2.0 mmol/L), and cell viability was tested using the MTT assay and ATP assay. As shown in Figures 1(a) and 1(b), the viability of HUVECs was reduced with increasing H_2_O_2_ concentrations compared with that of control cells (*p* < 0.05). Apoptosis of HUVECs treated with different concentrations of H_2_O_2_ (0, 0.5, and 1.0 mmol/L) was evaluated by performing flow cytometry to determine whether H_2_O_2_-induced apoptosis contributed to cell viability variation (Figures 1(c) and 1(d)). The results demonstrated that H_2_O_2_ induced apoptosis in a dose-dependent manner (*p* < 0.05).

**Figure 2:**
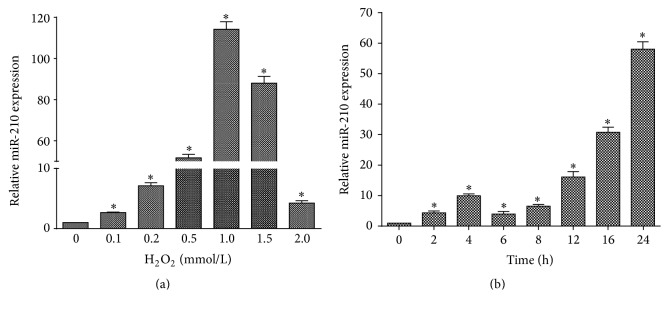
H_2_O_2_ increased miR-210 expression. (a) Incubation with H_2_O_2_ at different concentrations (0.1, 0.2, 0.5, 1.0, 1.5, and 2.0 mmol/L) for 24 h increased miR-210 expression compared that in untreated control cells. miR-210 expression was highest at 1.0 mmol/L. (b) HUVECs were treated with H_2_O_2_ at 0.5 mmol/L for 2, 4, 6, 8, 12, 16, and 24 h. miR-210 expression increased in all cases compared to that in untreated control cells. All *n* = 3, ^*∗*^*p* < 0.05.

We then tested the expression of miR-210 in HUVECs with qPCR in response to stimulation with H_2_O_2_ at different concentrations (0, 0.1, 0.2, 0.5, 1.0, 1.5, and 2.0 mmol/L). miR-210 expression increased (*p* < 0.05; Figure 2(a)), with the highest value observed at 1.0 mmol/L. In addition, we investigated the dependence of miR-210 expression on duration of H_2_O_2_ stimulation. In HUVECs treated with H_2_O_2_ at 0.5 mmol/L for 0, 2, 4, 6, 8, 16, and 24 h, miR-210 expression increased (*p* < 0.05; Figure 2(b)). These results indicated that miR-210 was involved in HUVEC apoptosis induced by H_2_O_2_.

### 3.2. The Effect of miR-210 on HUVEC Apoptosis in Response to H_2_O_2_

**Figure 3:**
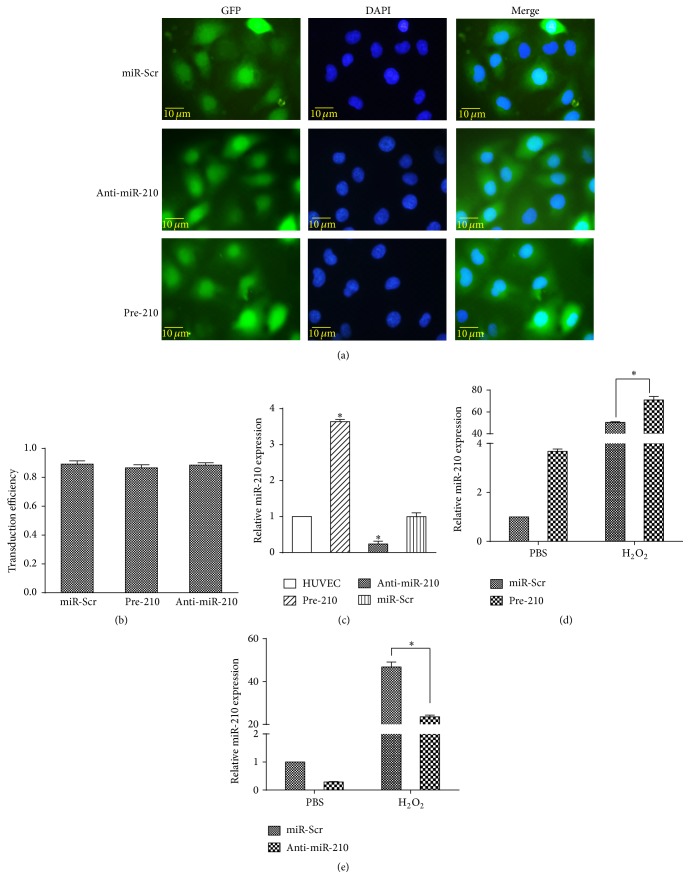
miR-210 expression variation in HUVECs transduced with lentivirus. (a) DAPI staining of HUVECs transduced with pre-210, anti-miR-210, and miR-Scr. (b) Quantification of transduction efficiency using the Image J software. (c) qRT-PCR results showing transgenic expression of miR-210 in HUVECs transduced with the lentivirus. (d) qRT-PCR results showing increased relative miR-210 expression in pre-210 HUVECs compared with miR-Scr HUVECs upon H_2_O_2_ treatment. (e) qRT-PCR results showing decreased relative miR-210 expression in anti-miR-210 HUVECs compared with miR-Scr HUVECs upon H_2_O_2_ treatment. All *n* = 3, ^*∗*^*p* < 0.05.

Since miR-210 expression increased in HUVECs treated with H_2_O_2_, we tested the effect of miR-210 on HUVECs apoptosis upon H_2_O_2_ treatment using gain-and-loss-of-function approaches. HUVECs were transduced with lentiviral vectors and observed under a fluorescence microscope (Figure 3(a)). The Image J software was used to quantitate the transduction efficiency (Figure 3(b)). The mean transduction efficiency of miR-Scr, pre-210, and anti-miR-210 was 0.88, 0.89, and 0.86, respectively. qRT-PCR showed that miR-210 expression levels were increased in the pre-210 cell line and decreased in the anti-miR-210 cell line compared with that in normal HUVECs (all *p* < 0.05). The level of miR-210 in the control cell line transfected with miR-Scr did not differ from that in normal HUVECs (Figure 3(b)). Moreover, during H_2_O_2_ stimulation (0.5 mmol/L, 24 h), miR-210 expression increased in the pre-210 cells and decreased in the anti-miR-210 cells compared with the miR-Scr cells (Figures 3(c) and 3(d)) (all *p* < 0.05).

**Figure 4:**
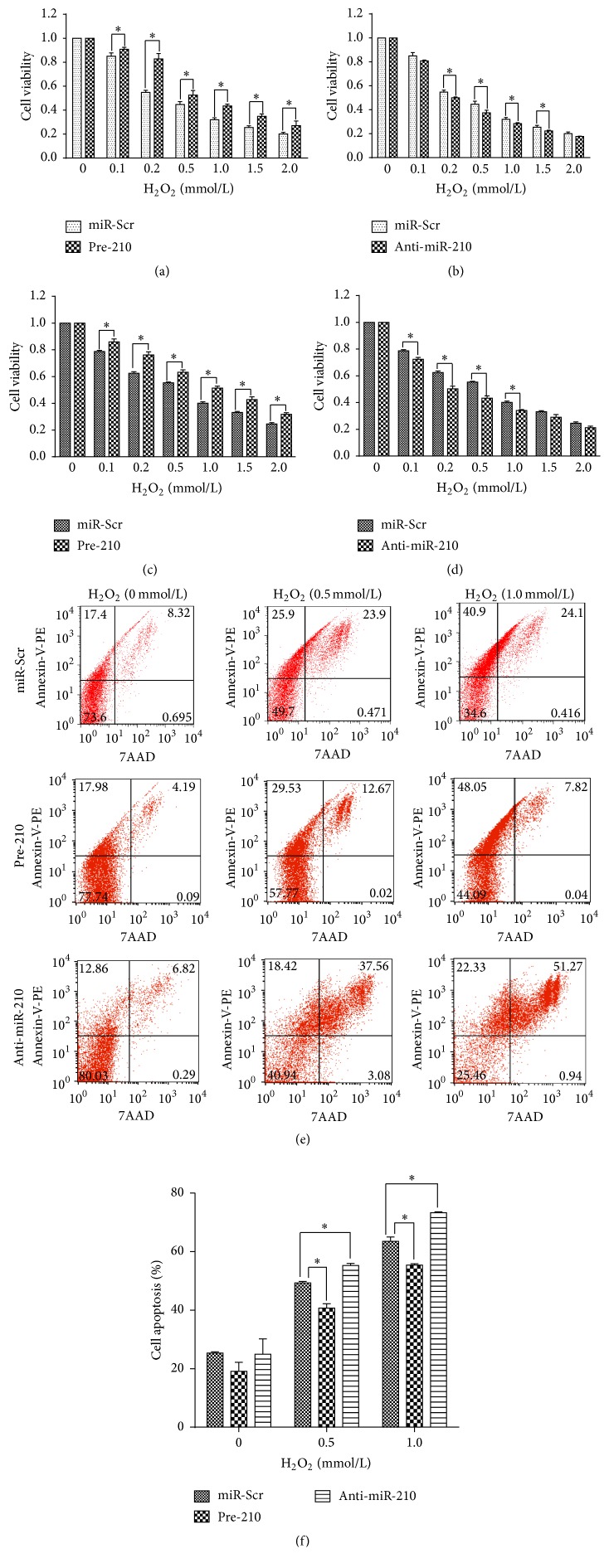
miR-210 reduced cell death in HUVECS treated with H_2_O_2_. (a) MTT assay results showing that miR-210 overexpression increased cell viability compared with that of miR-Scr HUVECs upon H_2_O_2_ treatment. (b) MTT assay results showing that miR-210 inhibition decreased cell viability compared with that of miR-Scr HUVECs upon H_2_O_2_ treatment. (c) ATP assay showing that miR-210 overexpression increased cell viability compared with that of miR-Scr HUVECs upon H_2_O_2_ treatment. (d) ATP assay showing that miR-210 inhibition decreased cell viability compared with that of miR-Scr HUVECs upon H_2_O_2_ treatment (e) Flow cytometry of the three transduced lines of HUVECs treated with H_2_O_2_ (0.5 mmol/L, 24 h). (f) Flow cytometry analysis showing that miR-210 protected HUVECs from apoptosis during H_2_O_2_ treatment. All *n* = 3, ^*∗*^*p* < 0.05.

We then investigated the effect of miR-210 on survival by treating HUVECs transduced with pre-210, anti-miR-210, and miR-Scr with H_2_O_2_ at different concentrations. The MTT assay showed that the cell viability ratio increased in pre-210 HUVECs at all H_2_O_2_ concentrations and decreased in anti-miR-210 HUVECs treated with H_2_O_2_ at 0.2, 0.5, 1.0, and 1.5 mmol/L compared with miR-Scr HUVECs (Figures 4(a) and 4(b)). Similar results were obtained with the ATP assay. Under H_2_O_2_ treatment, cell viability also increased in pre-210 HUVECs at all concentrations and decreased in anti-miR-210 HUVECs at 0.1, 0.2, 0.5 and 1.0 mmol/L compared with miR-Scr HUVECs (Figures 4(c) and 4(d)). In agreement, flow cytometry showed that the cell apoptosis ratio decreased in pre-miR-210 cells and increased in anti-miR-210 cells upon treating with H_2_O_2_ at 0.5 and 1.0 mmol/L (*p* < 0.05; Figures 4(c) and 4(d)).

### 3.3. The Effect of miR-210 on ROS Generation in HUVECs in Response to H_2_O_2_

**Figure 5:**
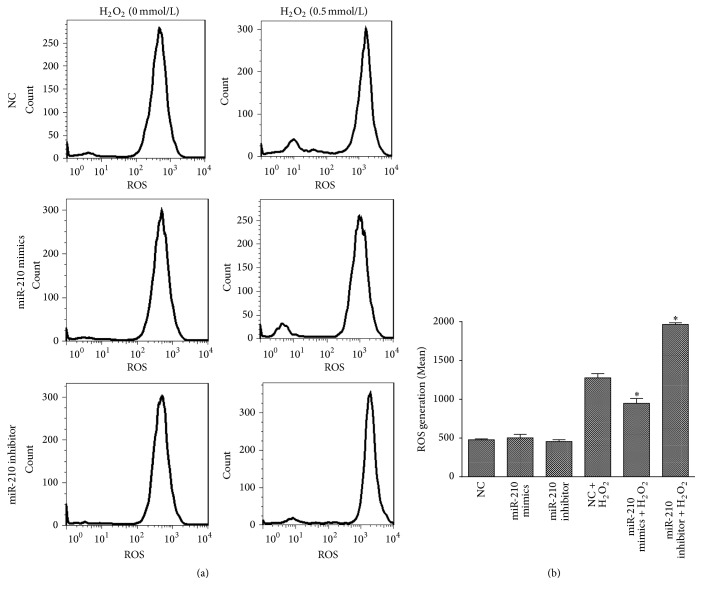
miR-210 reduced ROS generation in HUVECS treated with H_2_O_2_. (a) HUVECs were transfected with miR-210 mimics, miR-210 inhibitor, or NC and subsequently treated with H_2_O_2_ for 24 h (0, 0.5 mmol/L). Cells were collected and incubated with 10 *μ*M DCFH-DA for 20 minutes according to the manufacturer's instructions. After the incubation, the cells were washed with warm PBS, and ROS levels were analyzed by flow cytometry. (b) Quantification of mean fluorescent intensity in each group. Stars above bars refer to *p* values between NC-transfected cells treated with H_2_O_2_ and the corresponding group. All *n* = 3, ^*∗*^*p* < 0.05.

To investigate the effect of miR-210 on ROS generation, the cells were transfected with miR-210 mimics, miR-210 inhibitor, or NC, and ROS levels were determined after H_2_O_2_ treatment (0, 0.5 mmol/L, 24 h). Based on the results of qPCR (see S1 Fig in Supplementary Material available online at https://doi.org/10.1155/2017/3565613), miR-210 expression increased after transfection with miR-210 mimics and decreased after transfection with the miR-210 inhibitor compared with that of untransfected HUVECs. No difference was found between untransfected HUVECs and those transfected with the NC. ROS levels (Figure 5) increased in the NC group and were higher in the miR-210 inhibitor-transfected cells upon H_2_O_2_ treatment. Overexpression of miR-210 decreased the ROS levels during H_2_O_2_ treatment when compared with cells transfected with the NC.

### 3.4. H_2_O_2_ Effect on Relative Expression of Genes of the CASP8AP2 Pathway in HUVECs

**Figure 6:**
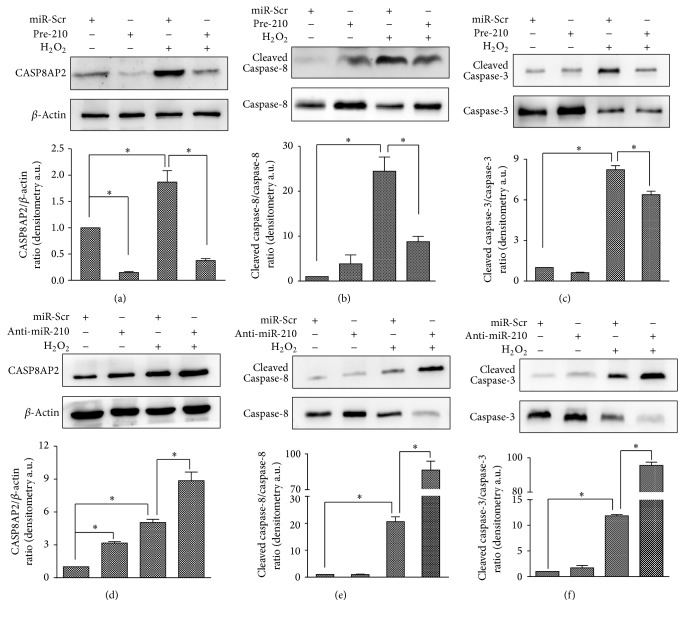
miR-210 downregulated CASP8AP2 pathway-related genes at the level of protein expression. (a) A Western blot showing that CASP8AP2 protein levels increased upon H_2_O_2_ treatment. miR-210 overexpression decreased the expression of CASP8AP2 in both H_2_O_2_-treated and untreated cells. (b) A Western blot showing that the cleaved caspase-8/caspase-8 ratio increased upon H_2_O_2_ treatment. miR-210 overexpression decreased the cleaved caspase-8/caspase-8 ratio upon H_2_O_2_ treatment. (c) A Western blot showing that the cleaved caspase-3/caspase-3 ratio increased upon H_2_O_2_ treatment. miR-210 overexpression decreased the cleaved caspase-3/caspase-3 ratio upon H_2_O_2_ treatment. (d) A Western blot showing that miR-210 inhibition increased CASP8AP2 protein levels in both the treated and untreated groups. (e) A Western blot showing that miR-210 inhibition increased the cleaved caspase-8/caspase-8 ratio upon H_2_O_2_ treatment. (f) A Western blot showing that miR-210 inhibition increased the cleaved caspase-3/caspase-3 ratio upon H_2_O_2_ treatment. All *n* = 3, ^*∗*^*p* < 0.05.

Since the CASP8AP2 pathway plays an important role in apoptosis, we investigated its contribution to the H_2_O_2_-induced apoptosis in HUVECs. The miR-Scr cells were treated with H_2_O_2_ at 0 or 0.5 mmol/L for 24 h, and expression of genes of the CASP8AP2 pathway was detected. There were no differences in mRNA expression after the H_2_O_2_ treatment (S2 Fig). However, Western blot results showed that, at protein level, CASP8AP2 expression increased after H_2_O_2_ treatment compared to the control (*p* < 0.05). In agreement, the cleaved caspase-8/caspase-8 and cleaved caspase-3/caspase-3 ratios were also increased (all *p* < 0.05) (Figures 6(a)–6(c)). These results indicated that the CASP8AP2 pathway was involved in HUVEC apoptosis during H_2_O_2_ treatment.

### 3.5. The Effect of miR-210 on the Expressions of CASP8AP2 Apoptosis Pathway-Related Genes in HUVECs Stimulated with H_2_O_2_

Since the CASP8AP2 pathway was involved in H_2_O_2_-mediated apoptosis, we investigated whether an association of the anti-apoptotic effect of miR-210 with CASP8AP2 pathway regulation existed. In HUVECs transduced with pre-210, anti-miR-210, and miR-Scr under H_2_O_2_ treatment (0 and 0.5 mmol/L, 24 h), no differences were found in the mRNA expression of CASP8AP2, caspase-8, or caspase-3 (S2 Fig). Nevertheless, protein expression of these genes differed in the three cell lines. CASP8AP2 expression was decreased in the pre-210 cells and increased in the anti-miR-210 cells compared with the miR-Scr cells (in cells both treated and not treated with H_2_O_2_, all *p* < 0.05; Figures 6(a) and 6(d)). Without H_2_O_2_ treatment, there were no differences in the caspase-8 cleavage or caspase-3 cleavage in the pre-210, anti-miR-210, and miR-Scr cells. However, under H_2_O_2_ stimulation, both caspase-8 cleavage and caspase-3 cleavage decreased in the pre-210 cells and increased in the anti-miR-210 cells versus the miR-Scr cells (all *p* < 0.05; Figures 6(b), 6(c), 6(e), and 6(f)). These results revealed that miR-210 downregulated the expression of CASP8AP2 pathway-related proteins.

## 4. Discussion

Endothelial cell apoptosis under oxidative stress plays a critical role in the initiation and progression of atherosclerosis [17]. In this study, we found that H_2_O_2_ induced HUVEC apoptosis through CASP8AP2 pathway activation. In addition, H_2_O_2_ stimulation upregulated the expression of miR-210, which protected HUVECs against H_2_O_2_-induced apoptosis by reducing ROS generation and affecting the CASP8AP2 pathway.

We found that H_2_O_2_ induced HUVEC apoptosis in a dose-dependent manner. In addition, CASP8AP2 pathway-related proteins were upregulated by the H_2_O_2_ treatment, which further contributed to the process of apoptosis. CASP8AP2 is a member of the apoptosis signaling complex that activates caspase-8, which results in the activation of caspase-3, eventually leading to apoptosis [5, 19]. In agreement, our results showed that the CASP8AP2 protein expression level increased in miR-Scr HUVECs treated with H_2_O_2_. Furthermore, we found that the cleaved caspase-8/caspase-8 and cleaved caspase-3/caspase-3 ratios increased, reflecting elevated activation of the initiator and effector caspases (caspase-8 and caspase-3, resp.) that intensified apoptosis. Therefore, activation of the CASP8AP2 pathway leads to apoptosis in HUVECs under oxidative stress.

Moreover, miR-210 was upregulated in HUVECs during H_2_O_2_ stimulation. Some studies, including our previous work, showed that miR-210 upregulation had an antiapoptotic effect in the rat H9C2 cell line [9], human mesenchymal stem cells [20], and mouse myoblast C2C12 cell line [21]. However, other studies showed that miR-210 had a proapoptotic effect in pulmonary artery epithelial cells [12], human lung cancer cell line [13], and esophageal cancer cell line [22]. Of interest, previous study demonstrated that miR-210 played different roles in HCT116 and MCF7 cells at different conditions [23]. In particular, miR-210 played a proapoptotic role at normal oxygen levels and an antiapoptotic role under hypoxia [23]. Our study found that miR-210 protected HUVECs against H_2_O_2_-induced apoptosis. In line with our results, Fasanaro et al. reported that miR-210 also had a prosurvival effect on HUVECs under hypoxia [6].

Furthermore, we found a negative correlation between miR-210 level and ROS generation upon H_2_O_2_ treatment. Increased ROS production is known to accelerate cell impairment. Our results indicated that miR-210 protected cells against apoptosis, which could be attributed to reduced ROS production under oxidative stress. Similar results were found in cardiomyocytes [4], mesenchymal stem cells [11], and ischemic hindlimbs [21]. However, contrasting results were obtained in colorectal cancer cells [24] and adipose-derived stem cells [25]. These differences could be due to the different cellular contexts. The underlying molecular mechanism of the reduced ROS generation could be related to the regulation of the c-Met pathway [11] and the expression of iron-sulfur cluster scaffold homolog [23].

We found that the antiapoptotic effect of miR-210 was also associated with the CASP8AP2 pathway. Overexpression of miR-210 inhibited the protein expression level of CASP8AP2 and thus decreased the cleaved caspase-8/caspase-8 ratio during H_2_O_2_ treatment, while miR-210 inhibition led to the opposite effect. These results suggested that miR-210 downregulated the CASP8AP2 pathway under oxidative stress.

Although CASP8AP2 was identified as a target of miR-210 in bone marrow-derived mesenchymal stem cells under anoxia [5], Barile et al. found no association between miR-210 and CASP8AP2 in HL-1 cells [26], which indicates that the target genes of miR-210 may vary with different cell types and conditions. Moreover, numerous studies identified several other target genes of miR-210, including* Efna3* [27], neuronal pentraxin 1 [28],* E2f3* [29],* PTPN2* [30], and* ptp1b* [10], suggesting that miR-210 has diverse roles in angiogenesis, DNA repair, cell cycle, migration, and apoptosis. Further studies on the association between miR-210 and other potential target genes in HUVECs under oxidative stress need to be conducted.

Of note, we found no differences in mRNA expression of CASP8AP2, caspase-8, and caspase-3 during H_2_O_2_ treatment versus control cells. It is possible that alterations in mRNA expression occurred prior to the changes in protein levels. Our results indicate that miR-210 protects HUVECs against oxidative stress in in vitro models. However, further research is needed to investigate the role of miR-210 in in vivo models and determine the underlying mechanisms.

## 5. Conclusions

We observed that miR-210 expression increased in HUVECs treated with H_2_O_2_. Moreover, miR-210 played a prosurvival and antiapoptotic role in HUVECs under oxidative stress by reducing ROS generation and downregulating the CASP8AP2 pathway. However, apoptosis-related pathways other than the CASP8AP2 pathway are known to exist. Further research on the relationship between miR-210 and these pathways is warranted. Our result may potentially lead to a new strategy for protecting against endothelial injury in atherosclerosis.

## Supplementary Material

Supplementary Figure 1. miR-210 expression in HUVECs transfected with miR-210 Mimics, miR-210 inhibitor, and NC. Supplementary Figure 2. Changes in CASP8AP2 pathway-related gene expression at the mRNA level in HUVECs after H_2_O_2_ treatment.
