# Supplementary material for: Protection of Human Umbilical Vein Endothelial Cells against Oxidative Stress by MicroRNA-210
Source: Oxid Med Cell Longev. 2017 Mar 7;2017:3565613. doi: 10.1155/2017/3565613 (PMC5359453; doi:10.1155/2017/3565613)
Supplement: Supplementary file 1 — Supplementary Figure 1. miR-210 expression in HUVECs transfected with miR-210 Mimics, miR-210 inhibitor, and NC. Supplementary Figure 2. Changes in CASP8AP2 pathway-related gene expression at the mRNA level in HUVECs after H2O2 treatment. [file 3565613.f1.pdf]

1

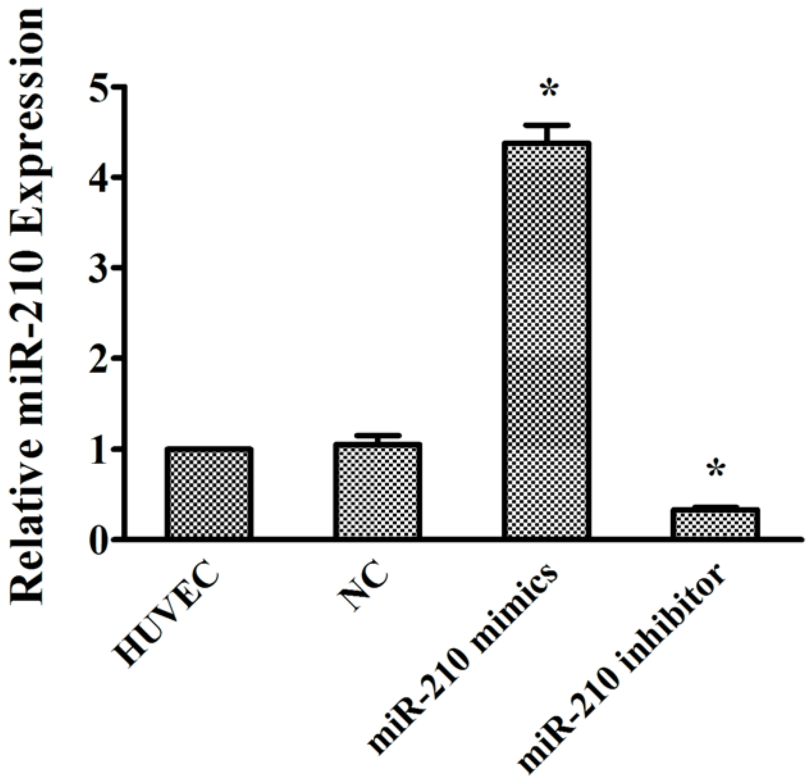

Relative miR-210 Expression
5
4
3
2
1
0
*
*
HUVEC
NC
miR-210 mimics
miR-210 inhibitor

2A

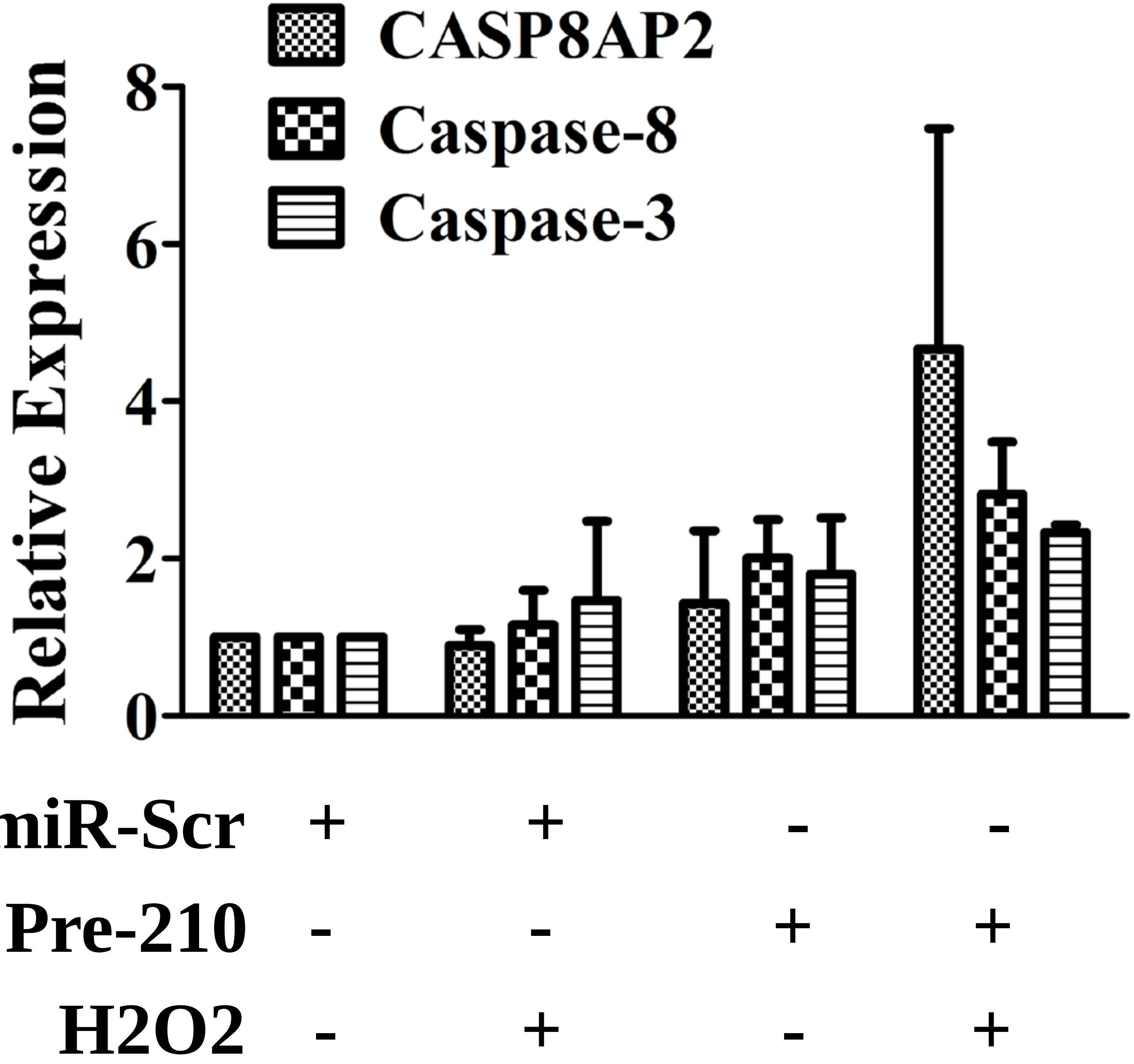
CASP8AP2
Caspase-8
Caspase-3
Relative Expression
8
6
4
2
0
miR-Scr + + - -
Pre-210 - - + +
H2O2 - + - +

2B

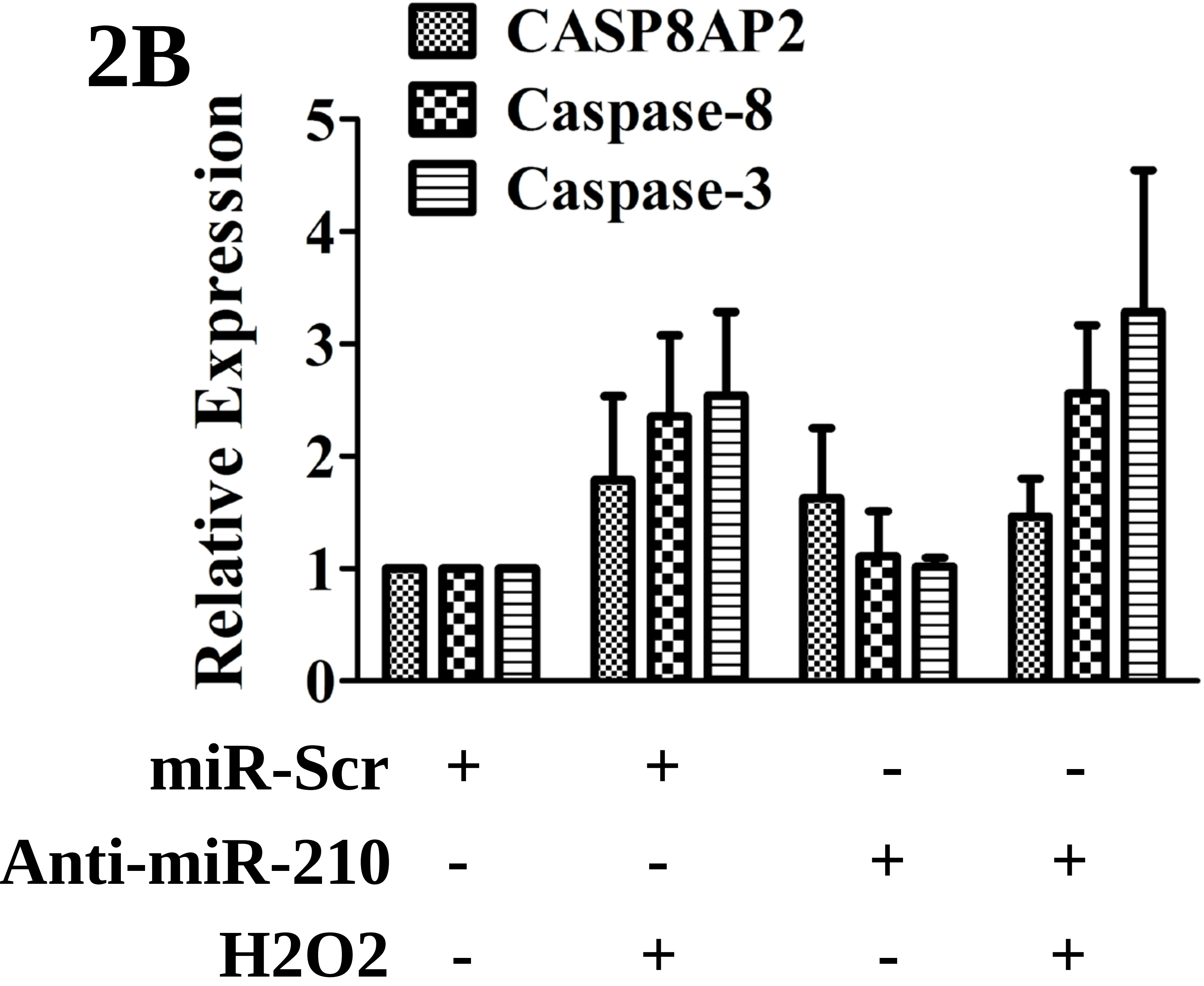
CASP8AP2
Caspase-8
Caspase-3
Relative Expression
0
1
2
3
4
5
miR-Scr + + - -
Anti-miR-210 - - + +
H2O2 - + - +

Supporting Information

S1 Fig. miR-210 Expression in HUVECs Transfected with miR-210 Mimics, miR-210 Inhibitor, and NC. qRT-PCR results showing that miR-210 expression increased in cells transfected with miR-210 mimics and decreased in cells transfected with the miR-210 inhibitor when compared with untransfected HUVECs. No difference in miR-210 expression was found between cells transfected with NC and untransfected HUVECs. All $n = 3$, $*p < 0.05$.

S2 Fig. Changes in CASP8AP2 Pathway-Related Gene Expression at the mRNA Level in HUVECs after H_2O_2 Treatment. (**A**) No differences were observed in the mRNA expression of CASP8AP2, caspase-8, and caspase-3 in miR-Scr HUVECs after H_2O_2 treatment compared with the control. Similarly, no differences were observed in the mRNA expression of CASP8AP2, caspase-8, and caspase-3 between pre-210 HUVECs and miR-Scr HUVECs upon H_2O_2 treatment. (**B**) No differences were present in the mRNA expression of CASP8AP2, caspase-8, and caspase-3 between anti-miR-210 HUVECs and miR-Scr HUVECs upon H_2O_2 treatment. All $n = 3$.
